# Brain Metastasis in a Young Patient: Consider the Rectum

**DOI:** 10.7759/cureus.20055

**Published:** 2021-11-30

**Authors:** Abdullah S Shaikh, Ravi Pavurala, Eric Gou

**Affiliations:** 1 Internal Medicine, University of Texas Medical Branch, Houston, USA; 2 Gastroenterology and Hepatology, University of Texas Medical Branch, Galveston, USA; 3 Gastroenterology, University of Texas Medical Branch, Galveston, USA

**Keywords:** brain tumor, brain, metastatic colo-rectal cancer, metastatic, colorectal cancer

## Abstract

Approximately 20% of patients with newly diagnosed colorectal cancer present with distant metastatic disease. Brain metastasis from colorectal cancer is uncommon and usually associated with metachronous metastases in other organs. We describe a rare case of a 49-year-old patient presenting with headaches and left-sided weakness found to have a solitary brain metastasis from primary rectal cancer. Primary rectal cancer, young age, lung and liver metastases, and KRAS mutation are risk factors associated with brain metastases in patients with colorectal cancer. Intracranial imaging should be considered as part of the workup in the staging of colorectal cancer in patients who are at high risk of brain metastasis.

## Introduction

Colorectal cancer is the third most common cancer in the United States [1] and while an increasing number of patients are now being diagnosed through colorectal cancer screening, the overwhelming majority of patients are still being diagnosed after the onset of symptoms [2, 3]. Typical presenting symptoms include a change in bowel habits, rectal bleeding, a rectal or abdominal mass, or iron deficiency anemia [4]. Moreover, approximately 20% of patients with newly diagnosed colorectal cancer present with distant metastatic disease [5]. The most common sites of metastasis are liver, lung, peritoneum and lymph nodes [6]. Brain metastasis from colorectal cancer is uncommon and usually associated with metachronous metastases in other organs. We describe a rare case of a 49-year-old patient presenting with headaches and left-sided weakness found to have a solitary brain metastasis from primary rectal cancer.

## Case presentation

**Figure 1:**
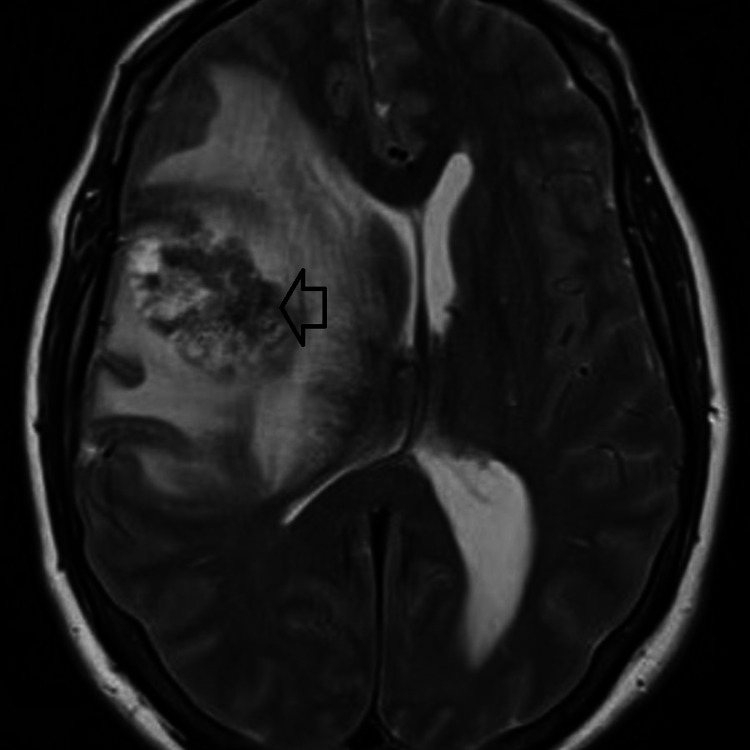
MRI of brain with and without contrast showing right lateral frontal intra-axial heterogenous enhancing mass measuring 4.4 x 4.0 x 3.7 cm with surrounding edema and right to left midline shift

A 49-year-old Caucasian male with a history of tobacco and alcohol abuse presented with worsening headaches, unintentional weight loss, and left-hand weakness. He reported a one-month history of worsening and persistent headaches as well as 30 lbs. of unintentional weight loss. He then developed left hand weakness approximately 2 weeks prior to presentation; he also reported increased left hand dyscoordination, which led him to dropping things often. He was taken to the emergency room by a coworker when he became unresponsive. His neurological exam on presentation was significant for 4/5 strength as well as a subtle drift in his left upper extremity. The rest of his physical exam was unremarkable. MRI brain showed a 4.4 x 4.0 x 3.7 cm right frontal mass with associated 1.3 cm right to left midline shift (Figure 1); the patient did not undergo positron emission tomography (PET) scan.

**Figure 2:**
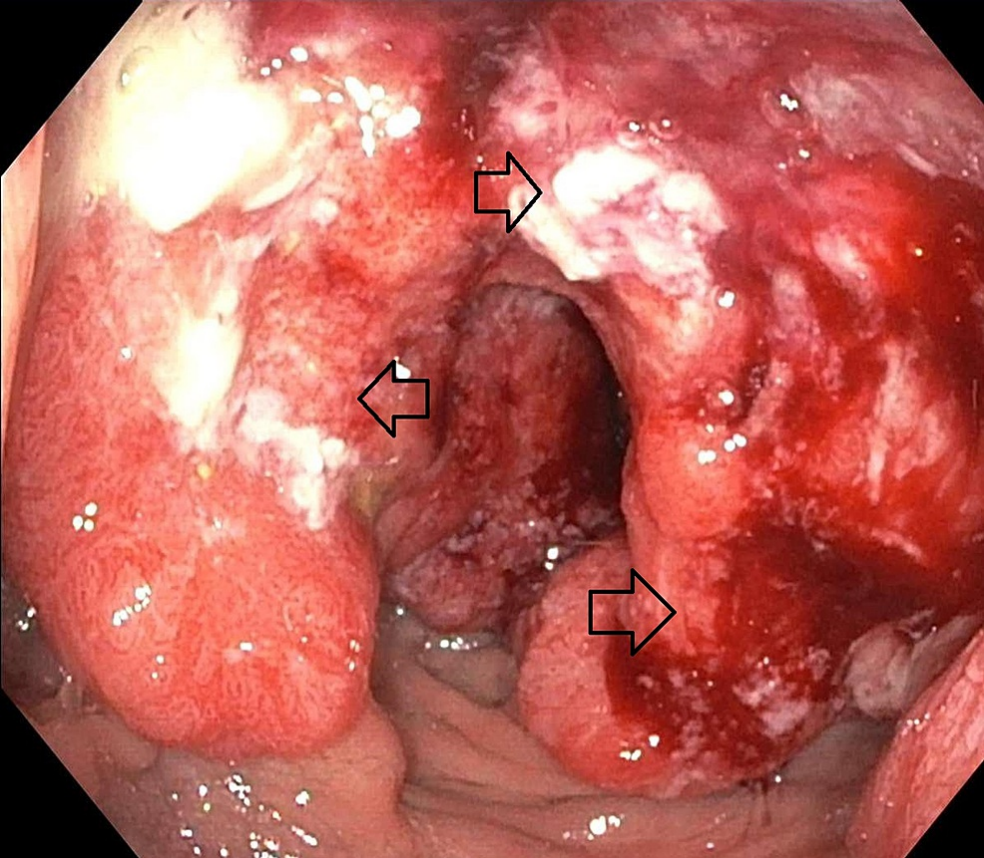
Endoscopic view of rectum showing fungating and polypoid circumferential mass with luminal narrowing extending into the anus

The patient was admitted to the neurosurgery service and underwent a right craniotomy due to initial suspicion of a high-grade glioma. However, pathology of the resected mass showed adenocarcinoma with an immunohistochemical profile most consistent with colorectal primary malignancy. Further review of systems revealed eight months of non-bloody diarrhea associated with fecal urgency. He did not have a family history of colorectal cancer in any first-degree relative. Carcinoembryonic antigen was elevated at 4.6 ng/mL. CT imaging of the chest, abdomen and pelvis showed ill-defined, nodular wall thickening of the rectum and sigmoid colon, sub-centimeter presacral and lower para-aortic lymph nodes and no other distant metastatic lesions. Flexible sigmoidoscopy demonstrated a partially obstructing rectal mass (Figure 2) with biopsies consistent with rectal adenocarcinoma. Immunohistochemistry showed microsatellite stability and molecular studies for KRAS, NRAS, and BRAF mutations were negative. He was referred to oncology and a repeat MRI brain did not show any evidence of any new intracranial metastases. The patient is awaiting systemic palliative chemotherapy with FOLFOX and bevacizumab.

## Discussion

The median age of diagnosis of rectal cancer is 63 years for both men and women with only 12% of cases of colorectal cancer occurring in patients under the age of 50 [7]. Moreover, the likelihood of a rectal cancer having a single metastasis to the nervous system is approximately 3% compared to 81% likelihood of a single metastasis to the liver (62%) or thorax (19%) as based on a multivariable logistic regression analysis of approximately 3,000 patients with rectal cancer with a single metastasis [6]. Another systematic review found that the incidence of a brain metastasis from colorectal cancer is extraordinarily uncommon with an incidence of approximately 0.6 to 3.2% and is usually an indicator of late-stage colorectal cancer [8]. Brain metastasis is much more common in other cancers such as lung cancer, breast cancer, testicular cancer, and melanoma with an incidence ranging from 10-50% depending on the type of cancer [8].

Our patient’s presentation is unique in that he presented at a young age with rectal cancer and a solitary metastasis to the brain. The incidence of brain metastasis at the initial diagnosis of colorectal cancer is 1.38% with a median survival of four months [9]. Moreover, a recent metanalysis and systemic review of brain metastasis in colorectal cancer recommended MRI imaging of the brain if a patient with colorectal cancer had any neurological symptoms or risk factors for brain metastases such as the presence of lung metastases, KRAS mutation, high carcinoembryonic antigen (CEA) level, or rectal cancer [10]. The rising incidence of early-onset colorectal cancer, which are characterized by poorer cell differentiation, more advanced stage at diagnosis, as well as more often having a left-sided colon primary tumor [11], is one of the primary reasons the U.S. Preventative Task Force updated their recommendation to start colorectal cancer screening from age 45 [12], instead of waiting till age 50.

There is an increasing concern of microsatellite instability leading to proximal colon cancer with RAS and BRAF mutations found in approximately 52% [13] and 10% [14] of colorectal cancer, respectively. While our patient did not have evidence of microsatellite instability or a proximal colon cancer, RAS and BRAF mutation testing has been shown to have prognostic and predictive value in metastatic colorectal cancer. Treatment with anti-epidermal growth factor receptor (EGFR) antibodies has been shown to be less effective in patients with KRAS mutations [15] and patients with metastatic colorectal cancer and either KRAS or BRAF mutations have been shown to have worsened outcomes [16].

## Conclusions

In conclusion, brain metastases are the most common intracranial neoplasm and typically arise from melanoma, lung, and breast cancers. Brain metastases from gastrointestinal cancers are uncommon and occur in less than 4% of patients with colorectal cancer. Primary rectal cancer, young age, lung and liver metastases, and KRAS mutation are risk factors associated with brain metastases in patients with colorectal cancer. Intracranial imaging should be considered as part of the workup in the staging of colorectal cancer in patients who are at high risk of brain metastasis.
